# Factors for short-term outcomes in patients with a minor stroke: results from China National Stroke Registry

**DOI:** 10.1186/s12883-015-0505-z

**Published:** 2015-12-09

**Authors:** Lingyun Wu, Anxin Wang, Xianwei Wang, Xingquan Zhao, Chunxue Wang, Liping Liu, Huaguang Zheng, Yongjun Wang, Yibin Cao, Yilong Wang, on behalf of the China National Stroke Registry investigators

**Affiliations:** Graduate School, North China University of Science and Technology, Tangshan, China; Department of Neurology, Beijing Tiantan Hospital, Capital Medical University, Beijing, China; China National Clinical Research Center for Neurological Diseases, Beijing, China; Center of Stroke, Beijing Institute for Brain Disorders, Beijing, China; Beijing Key Laboratory of Translational Medicine for Cerebrovascular Disease, Beijing, China; Department of Epidemiology and Health Statistics, School of Public Health, Capital Medical University, Beijing, China; Department of Neurology, Tangshan Gongren Hospital, Tangshan, China

**Keywords:** Minor stroke, Risk factors, Stroke recurrence, Poor functional outcome

## Abstract

**Background:**

Stroke recurrence and disability in patients with a minor stroke is one of the most depressing medical situations. In this study, we aimed to identify which factors were associated with adverse outcomes of a minor stroke.

**Methods:**

The China National Stroke Registry (CNSR) is a nationwide prospective registry for patients presented to hospitals with acute cerebrovascular events between September 2007 and August 2008. The 3-month follow-up was completed in 4669 patients with a minor stroke defined as the initial neurological severity lower than 4 in the National Institutes of Health Stroke Scale (NIHSS). Multivariate model was used to determine the association between risk factors and clinical outcomes.

**Results:**

Of 4669 patients with a minor stroke during 3-month follow-up, 459 (9.8 %) patients experienced recurrent stroke, 679 (14.5 %) had stroke disability and 168 (3.6 %) died. Multivariate model identified hypertension, diabetes mellitus, atrial fibrillation, coronary heart disease and previous stroke as independent predictors for the recurrent stroke. Age, diabetes mellitus, atrial fibrillation, previous stroke and time from onset to admission < 24 h were independent predictors for stroke disability. The independent predictors for the all-caused death were age, atrial fibrillation, and coronary heart disease.

**Conclusions:**

The short-term risk of poor clinical outcome in Chinese patients with a minor stroke was substantial. Therefore, patients with a minor stroke should be given expeditious assessment and urgent aggressive intervention.

## Background

Stroke recurrence and dependency in patients with an initial non-disabling stroke is one of the most depressing medical situations worldwide. Stroke has been the leading cause of death and acquired adult disability in China [1, 2], furthermore, where the incidence rate of ischemic stroke increased by 8.7 % annually on average from 1984 to 2004 [3]. Increasing evidences have suggested that a significant minority of patients with an acute non-disabling stroke become disabled or dead due to recurrent strokes or neurological deterioration within first few hours or days [4–6]. A population-based study showed that the true risk of recurrent stroke after minor stroke is up to about 18 % at 3 months [7]. As for stroke-related disability, it is relatively common among the patients with mild or rapid improving symptom who were not treated with the intravenous thrombolysis [5, 6, 8, 9].

Considering the variable clinical course in the immediate aftermath of a minor stroke, it will become more important to assess risk factors for detecting patients with a poorer clinical outcome, for whom aggressive prevention treatment like dual or triple antiplatelet is urgently needed. At present, majority of studies have been focusing on the early risk of recurrent stroke after transient ischemic attack (TIA) and a minor stroke [10–13], but data about the short-term risk of poor functional outcome and subsequent stroke assessed in patients with minor stroke was limited, and even not available in China. Using China National Stroke Registry (CNSR), we aimed to identify factors associated with early stroke recurrence, stroke disability and all-cause death after an initial minor stroke during a 90-day follow-up period.

## Methods

### Study population

The present cohort was from the China National Stroke Registry (CNSR), which is a nationwide prospective registry for patients presented to hospitals with acute cerebrovascular events between September 2007 and August 2008. The detailed design, rationale, and basic description of the CNSR have been published previously [14, 15]. We included patients in the current analysis if they presented with an ischemic stroke diagnosed according to World Health Organization criteria [16, 17] combined with the confirmation of brain computed tomography or magnetic resonance imaging. The minor stroke was defined as the initial neurological severity lower than 4 in the National Institutes of Health Stroke Scale (NIHSS) [17, 18]. We excluded the patients without follow-up information at 3 months after stroke onset. The study was sponsored by the Ministry of Science and Technology and the Ministry of Health of the People’s Republic of China and approved by the central institutional review board at Beijing Tiantan Hospital and local ethical committees at each participating hospital. Written informed consent for inclusion was signed by patients or legally authorized representatives.

### Data collection

All research coordinators and study investigators were trained and certified to assess National Institutes of Health Stroke Scale scores before the beginning of the study. We collected baseline information including patient demographics, vascular risk factors, stroke severity (NIHSS score at admission and 24 h later), stroke management, diagnosis and discharge status. Vascular risk factors included hypertension, diabetes mellitus, dyslipidemia, atrial fibrillation, coronary heart disease, previous stroke, current or previous smoking and moderate or heavy alcohol consumption (≥2 standard alcohol consumption per day) and body mass index (BMI) at admission. Hypertension was defined as systolic blood pressure ≥ 140 mmHg or diastolic blood pressure ≥ 90 mmHg, any use of antihypertensive drug, or self-reported history of hypertension. Diabetes mellitus was defined as fasting glucose level ≥ 7.0 mmol/L, non-fasting glucose concentration ≥ 11.1 mmol/L, any use of glucose-lowering drugs, or any self-reported history of diabetes. Dyslipidemia was defined as serum triglyceride ≥ 1.7 mmol/L, low-density lipoprotein cholesterol ≥ 3.6 mmol/L, high-density lipoprotein cholesterol ≤ 1.0 mmol/L, any use of lipid-lowering drugs, or any self-reported history of dyslipidemia. Atrial fibrillation was defined as history of atrial fibrillation confirmed by at least one electrocardiogram or presence of the arrhythmia during hospitalization. The treatment of atrial fibrillation defined the use of anticoagulation agents during hospitalization and after discharge. BMI was calculated by dividing measured weight in kilograms by the square of measured height in meters. In addition, neurological deterioration was considered as the increase of NIHSS ≥ 4 at 24 h after admission from baseline [19]. Etiologic subtypes of ischemic stroke were classified by the Stop Stroke Study Trial of Org 10172 in Acute Stroke Treatment (SSS-TOAST) classification criteria [20].

### Outcome assessment

The follow up was done by telephone interview. Trained research personnel making the phone calls were blinded to patients’ baseline clinical status. Patients were asked the standardized follow-up questions at 3 months after stroke onset. Outcomes data collected included stroke recurrence, stroke disability and all-cause death. Recurrent cerebrovascular events included ischemic stroke, intracranial hemorrhage and subarachnoid hemorrhage. Cases of recurrent strokes were cross-checked with the treating hospitals to ensure the accuracy of diagnosis. In case of a suspected stroke recurrence without hospitalization, the case was adjudicated by the trial executive committee. Stroke disability was defined as modified Rankin Scale (mRS) of 3–6 at 3 months after stroke onset. All-cause mortality was defined as death from any cause which was confirmed by either a death certificate from the local citizen registry or the record of the treating hospital. When no official documentation was available, case fatality was decided if death was reported on two consecutive follow-up periods by different proxies.

### Statistical analysis

Continuous variables are expressed as means with standard deviation (SD) or median with interquartile range (IQR), as appropriate. Categorical data are presented as proportions. The differences in baseline demographic and clinical features between patients with poor clinical outcomes (early recurrent stroke, stroke disability and all-cause death) and those without poor clinical outcomes at 3 months after minor stroke were tested for continuous variables with normal distribution using one-way analysis of variance and continuous variables with skewed distribution using Kruskal-Wallis test. The χ2 or Fisher exact test was used for categorical variables.

We analyzed the association between the clinical outcomes including early recurrent stroke, stroke disability and all-cause death and relevant covariates with logistic regression analysis adjusting age, gender, hypertension, diabetes mellitus, dyslipidemia, atrial fibrillation, coronary heart disease, previous stroke, current or previous smoking, moderate or heavy alcohol, BMI at admission, and admission within 24 h or not after stroke onset.

We have determined that two-tailed p values less than 0.05 was statistically significant. All statistical analyses were carried out with SAS Version 9.4 software. (SAS Institute Inc., U.S.)

## Results

### Patient recruitments

**Fig. 1:**
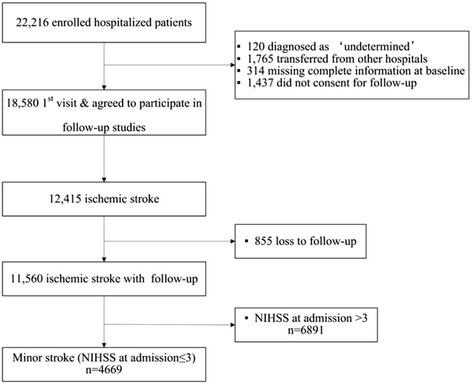
Flow chart showing the patient selection. NIHSS, National Institutes of Health Stroke Scale

Of the 22,216 patients enrolled in the CNSR, 18,580 patients had complete baseline information and agreed to participate in the 90-day follow-up. Among them, 855 patients were excluded due to loss to follow-up at 3 months. After excluding 6891 patients with an initial NIHSS ≥ 4, a total of 4669 patients with minor stroke (the initial NIHSS < 4) were remained finally (Fig. 1).

### Univariate analysis of patients with or without recurrent stroke, stroke disability and all-cause death at 3 months after minor stroke

Of 4669 patients with a minor stroke during 90-day follow-up, 459 (9.8 %) patients experienced recurrent stroke, 679 (14.5 %) had stroke disability and 168 (3.6 %) died. (Tables 1, 2 and 3) Of 168 patients who died, 77 (45.8 %) died of recurrent stroke. In univariate analysis, patients with stroke recurrence at 3 months after minor stroke had a higher rate of hypertension, diabetes mellitus, dyslipidemia, atrial fibrillation, coronary heart disease, previous stroke, time from onset to admission < 24 h, neurological deterioration and large-artery atherosclerosis/cardioembolism subtype of toast type than those without recurrence. Patients with recurrence stroke were more likely to be older and have a higher median NIHSS score at admission than those without recurrence. There was no difference between patients with recurrence stroke and those without recurrence stroke in gender, current or previous smoking, moderate or heavy alcohol, treated atrial fibrillation, median BMI at admission and median NIHSS score at 24 h (Table 1).

**Table 1:** Univariate analysis of patients with or without stroke recurrence at 3 months after minor stroke

Variables	Overall	Recurrence	No recurrence	*P* value
Sample size, n (%)	4669	459 (9.8)	4210 (90.2)	
Age, median (IQR), year	65 (55–74)	67 (57–76)	65 (55–73)	<0.001
Female, n (%)	1656 (35.5)	159 (34.6)	1497 (35.6)	0.70
Vascular risk factors				
Hypertension, n (%)	3438 (73.6)	365 (79.5)	3073 (73.0)	0.003
Diabetes mellitus, n (%)	1274 (27.3)	148 (32.2)	1126 (26.8)	0.01
Dyslipidemia, n (%)	550 (11.8)	68 (14.8)	482 (11.4)	0.03
Atrial fibrillation, n (%)	270 (5.8)	53 (11.6)	217 (5.2)	<0.001
Treated atrial fibrillation, n (%)	29 (10.7)	4 (7.5)	25 (11.5)	0.55
Coronary heart disease, n (%)	600 (12.8)	90 (19.6)	510 (12.1)	<0.001
Previous stroke, n (%)	1451 (31.1)	196 (42.7)	1255 (29.8)	<0.001
Current or previous smoking, n (%)	1872 (40.1)	178 (38.8)	1694 (40.2)	0.54
Moderate or heavy alcohol, n (%)	470 (10.1)	47 (10.2)	423 (10.0)	0.90
BMI at admission, median (IQR), kg/m^2^	24.22 (22.3–26.3)	24.22 (22.4–26.6)	24.22 (22.3–26.3)	0.41
NIHSS score, median (IQR)				
At admission	2 (1–3)	1 (0–2)	2 (1–3)	0.001
At 24 h	2 (1–3)	2 (1–3)	2 (1–3)	0.74
Time from onset to admission <24 h, n (%)	2472 (52.9)	263 (57.3)	2209 (52.5)	0.049
Neurological deterioration, n (%)	166 (3.6)	31 (6.8)	135 (3.2)	<0.001
TOAST subtype, n (%)				<0.001
Large-artery atherosclerosis	1951 (41.8)	208 (45.3)	1743 (41.4)	
Small-artery occlusion	1066 (22.8)	84 (18.3)	982 (23.3)	
Cardioembolism	131 (2.8)	28 (6.1)	103 (2.4)	
Other	176 (3.8)	13 (2.8)	163 (3.9)	

*IQR* interquartile range, *NIHSS* National Institutes of Health Stroke Scale, *BMI* body mass index, *TOAST* Trial of Org 10172 in Acute Stroke Treatment

**Table 2:** Univariate analysis of patients with or without stroke disability at 3 months after minor stroke

Variables	Overall	Disability	No disability	*P* value
Sample size, n (%)	4669	679 (14.5)	3990 (85.5)	
Age, median (IQR), year	65 (55–74)	72 (62–78)	64 (55–72)	<0.001
Female, n (%)	1656 (35.5)	281 (41.4)	1375 (34.5)	<0.001
Vascular risk factors				
Hypertension, n (%)	3438 (73.6)	527 (77.6)	2911 (73.0)	0.01
Diabetes mellitus, n (%)	1274 (27.3)	251 (37.0)	1023 (25.6)	<0.001
Dyslipidemia, n (%)	550 (11.8)	79 (11.6)	471 (11.8)	0.90
Atrial fibrillation, n (%)	270 (5.8)	75 (11.0)	195 (4.9)	<0.001
Coronary heart disease, n (%)	600 (12.8)	124 (18.3)	476 (11.9)	<0.001
Previous stroke, n (%)	1451 (31.1)	273 (40.2)	1178 (29.5)	<0.001
Current or previous smoking, n (%)	1872 (40.1)	229 (33.7)	1643 (41.2)	<0.001
Moderate or heavy alcohol, n (%)	470 (10.1)	39 (5.7)	431 (10.8)	<0.001
BMI at admission, median (IQR), kg/m2	24.22 (22.3–26.3)	24.03 (22.0–26.0)	24.22 (22.5–26.4)	0.03
NIHSS score, median (IQR)				
At admission	2 (1–3)	2 (0–3)	2 (1–3)	0.06
At 24 h	2 (1–3)	3 (2–3)	2 (1–3)	<0.001
Time from onset to admission <24 h, n (%)	2472 (52.9)	1641 (54.5)	831 (50.2)	0.005
Neurological deterioration, n (%)	166 (3.6)	270 (39.8)	1927 (48.3)	<0.001
TOAST subtype, n (%)				<0.001
Large-artery atherosclerosis	1951 (41.8)	303 (44.6)	1648 (41.3)	
Small-artery occlusion	1066 (22.8)	94 (13.8)	972 (24.4)	
Cardioembolism	131 (2.8)	44 (6.5)	87 (2.2)	
Other	176 (3.8)	21 (3.1)	155 (3.9)	

Stroke disability was defined as modified Rankin Scale >2; *IQR* interquartile range, *BMI* body mass index, *NIHSS* National Institutes of Health Stroke Scale, *TOAST* Trial of Org 10172 in Acute Stroke Treatment

**Table 3:** Univariate analysis of patients with or without all-cause death at 3 months after minor stroke

Variables	Overall	Death	No death	*P* value
Sample size, n (%)	4669	168 (3.6)	4501 (96.4)	
Age, median (IQR), year	65 (55–74)	72 (60–77)	65 (55–73)	<0.001
Female, n (%)	1656 (35.5)	70 (41.7)	1586 (35.2)	0.87
Vascular risk factors				
Hypertension, n (%)	3438 (73.6)	122 (72.6)	3316 (73.7)	0.76
Diabetes mellitus, n (%)	1274 (27.3)	46 (27.4)	1228 (27.3)	0.98
Dyslipidemia, n (%)	550 (11.8)	21 (12.5)	529 (11.8)	0.77
Atrial fibrillation, n (%)	270 (5.8)	25 (14.9)	245 (5.4)	<0.001
Coronary heart disease, n (%)	600 (12.9)	40 (23.8)	560 (12.4)	<0.001
Previous stroke, n (%)	1451 (31.1)	68 (40.5)	1383 (30.7)	0.007
Current or previous smoking, n (%)	1872 (40.1)	62 (36.9)	1810 (40.2)	0.39
Moderate or heavy alcohol, n (%)	470 (10.1)	11 (6.6)	459 (10.2)	0.12
BMI at admission, median (IQR), kg/m2	24.22 (22.3–26.3)	23.61 (21.7–26.0)	24.22 (22.4–26.3)	<0.001
NIHSS score, median (IQR)				
At admission	2 (1–3)	1 (0–2)	2 (1–3)	<0.001
At 24 h	2 (1–3)	2 (1–3)	2 (1–3)	0.006
Time from onset to admission <24 h, n (%)	2472 (52.9)	97 (57.7)	2375 (52.8)	0.20
Neurological deterioration, n (%)	166 (3.6)	19 (11.3)	147 (3.3)	<0.001
TOAST subtype, n (%)				<0.001
Large-artery atherosclerosis	1951 (41.8)	66 (39.3)	1855 (41.9)	
Small-artery occlusion	1066 (22.8)	19 (11.3)	1047 (23.3)	
Cardioembolism	131 (2.8)	15 (8.9)	116 (2.6)	
Other	176 (3.8)	5 (3.0)	171 (3.8)	

*IQR* interquartile range, *BMI* body mass index, *NIHSS* National Institutes of Health Stroke Scale, *TOAST* Trial of Org 10172 in Acute Stroke Treatment

Table 2 showed older and female patients were more likely to be dependency at 3 months after minor stroke (*P* < 0.05). Patients with dependency were significantly more likely to have hypertension, diabetes mellitus, atrial fibrillation, coronary heart disease , previous stroke, current or previous smoking, moderate or heavy alcohol, time from onset to arriving at hospital < 24 h, neurological deterioration and large-artery atherosclerosis/cardioembolism subtype of toast type, as compared with those without dependency. Finally, patients with dependency had a higher median BMI at admission and median NIHSS score at 24 h than those without recurrence. There was no statistically significant difference between the patients with dependency and those without dependency in the following factors: dyslipidemia and median NIHSS score at admission.

Factors associated with all-cause death in patients after minor stroke are showed in Table 3. In the univariate analysis, the variables associated with death include age, atrial fibrillation, coronary heart disease, previous stroke, median BMI and NIHSS score, neurological deterioration and TOAST subtype (all *P* value < 0.05).

### Predictors of outcomes

**Fig. 2:**
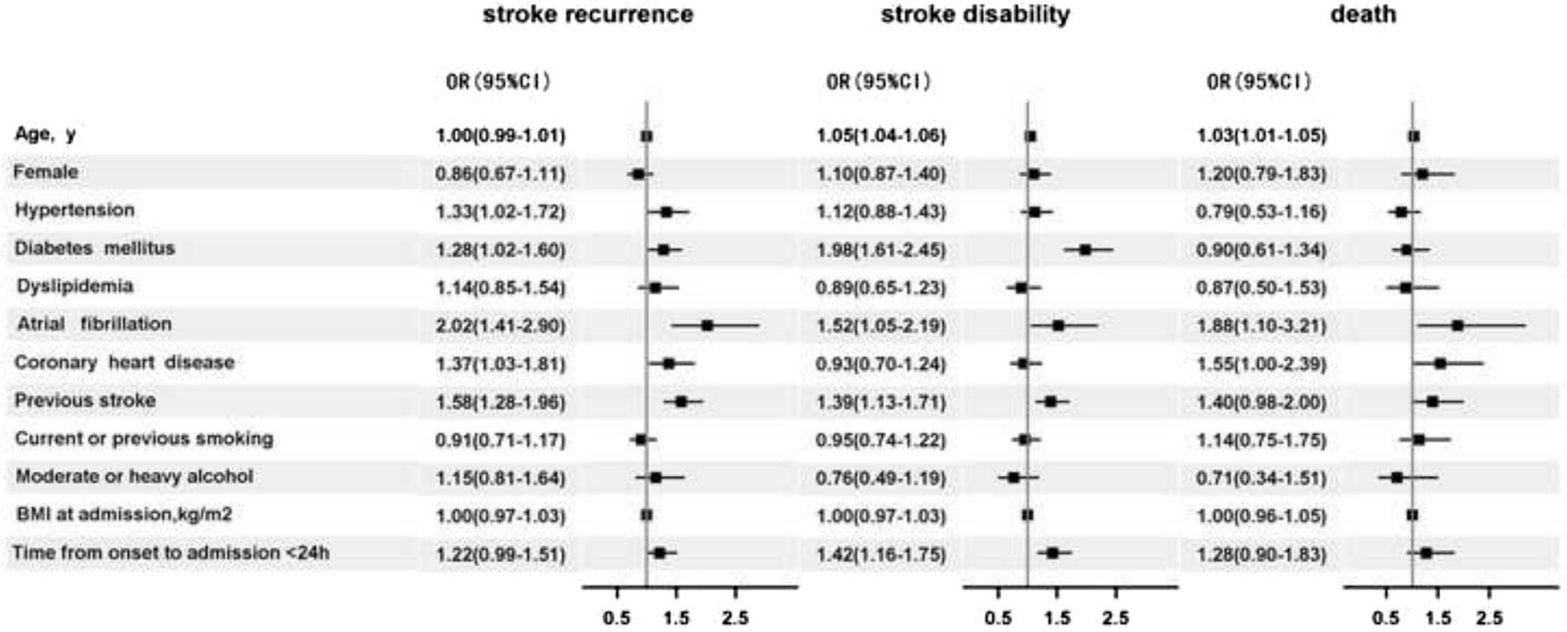
The adjusted ORs for stroke recurrence, disability and death at 3 months after stroke onset. Stroke disability was defined as modified Rankin Scale > =3.OR denotes Odds Ratio; CI, Confidence Internal; BMI, Body Mass Index. Adjusted for age, gender, hypertension, diabetes mellitus, dyslipidemia, atrial fibrillation, coronary heart disease, previous stroke, current or previous smoking, moderate or heavy alcohol, BMI at admission, and admission within 24 h

Figure 2 showed adjusted odds ratios (OR) and 95 % confidence internals (CI) of the risk factors for stroke recurrence, stroke disability and all-caused death at 3 months in patients with the minor stroke using the multivariable logistic regression analysis. We identified the hypertension, diabetes mellitus, atrial fibrillation, coronary heart disease and previous stroke as independent predictors for recurrent stroke at 3 months after stroke onset. The independent predictors for the stroke disability at 3 months were age, diabetes mellitus, atrial fibrillation, previous stroke and time from onset to admission < 24 h. Age, atrial fibrillation and coronary heart disease were independently associated with the all-caused death at 3 months after stroke onset.

## Discussion

In the largest sample of Chinese stroke adults to date, the present study found that a significant minority of patients with the minor stroke experienced permanent neurological deficits or death and the recurrent stroke during the 3-month follow-up period. Nevertheless, patients at earlier phase after minor stroke were more likely to have high risk of stroke recurrence and poor functional outcome at 3 months after stroke onset.

Our data showed a 3-month recurrence rate of 9.8 % in patients with a minor stroke. The short-term risk of stroke recurrence was consistent to about 10 % at 3 months after TIA [10, 11], but lower than the range between 14 and 18 % found in two previous studies of patients with a minor stroke [7, 21]. The early risk of sequent stroke is commonly underestimated with the reason that patients were usually enrolled several days after a minor stroke and any patient who had a major stroke before the recruitment was not included. We also detected that hypertension, diabetes mellitus and previous stroke were predictors of early recurrent stroke, as was suggested in previous studies [10, 11]. Atrial fibrillation was independently associated with the risk of early recurrent stroke, which was not confirmed in population from western countries [11, 21]. The possible explanation is that in clinical practice of China, unlike North America and Europe, only 14.3 % stroke patients with atrial fibrillation receive oral anticoagulation in real clinical practice [22]. Of those patients who developed recurrent stroke in our study, 7.5 % patients with atrial fibrillation received anticoagulation, which was lower than the early study. Previous studies had reported that coronary artery disease was strongly associated with stroke [23, 24]. Carotid atherosclerosis and stroke of carotid origin are coronary artery disease risk equivalents [25], thus coronary artery disease and cerebrovascular diseases are highly correlated. Our present study found that coronary artery disease was significantly associated with the risk of stroke recurrence at 3 months after adjusting other risk factors, so coronary artery disease can be regarded as an independent risk factor. The impact of time after a minor stroke for stroke recurrence was also dissected out in multivariate model analysis. It presented that the patients at the earlier phase of minor stroke were more likely to experience subsequent stroke (OR 1.22; 95 % CI, 0.99-1.51; *p* = 0.06).

The clinical functional outcomes of patients with minor stroke is not invariably benign. The rate of patients with stroke disability at 3 months in this study was up to 14.5 %. The multivariable-adjusted analysis revealed that patients at earlier phase of minor stroke (≤24 h) were about 1.5 times more likely to have a stroke disability at 3 months, and tended to have all-cause death at 3 months. The reason was unknown, but it was reported in a previous study [26] that patients with more severe stroke immediately contacted emergency services after stroke onset to avoid disability and death. Therefore, it’s likely that the time from onset to admission was a proxy for stroke severity even in minor strokes.

The study had some limitations. First, the participating hospitals were selected by convenience in nature. The majority of hospitals selected were tertiary hospitals that may not represent the status quo of stroke in rural and smaller community hospitals. Second, unavoidable delays before the inclusion of patients in the study led to the underestimation of the recurrent stroke risk and poor functional outcomes. The reason was that a portion of patients with minor stroke prior to study enrollment were not categorized into the group of minor stroke due to recurrent stroke or worsening of their presenting events. Third, it would be helpful if the abnormality on the imaging metric could be taken into as part of multivariate analysis for neurological deterioration. However, the detailed information about imaging was unavailable and the analysis couldn’t be performed. Furthermore, as we mainly focus on the impact of risk factors before admission on outcomes, the other potentially significant factors including treatments and therapies variables during hospitalization and at discharge were not included in the analysis.

## Conclusions

Our study indicated the short-term risk of early recurrent stroke and poor functional outcome in Chinese patients with a minor stroke was substantial. Therefore, patients with a minor stroke should be given expeditious assessment and urgent aggressive intervention.

## Abbreviations

BMI: body mass index
CI: confidence internals
CNSR: China National Stroke Registry
IQR: interquartile range
mRS: modified Rankin Scale
NIHSS: National Institutes of Health Stroke Scale
OR: odds ratios
SD: standard deviation
SSS-TOAST: Stop Stroke Study Trial of Org 10172 in Acute Stroke Treatment
TIA: transient ischemic attack
